# Retraction: Hirudin ameliorates immunoglobulin a nephropathy by inhibition of fibrosis and inflammatory response

**DOI:** 10.1080/0886022X.2025.2462380

**Published:** 2025-02-17

We, the Editors and Publisher of Renal Failure, have retracted the following article:

Deng F, Zhang J, Li Y, Wang W, Hong D, Li G, Feng J. Hirudin ameliorates immunoglobulin A nephropathy by inhibition of fibrosis and inflammatory response. Renal Failure. 2019;41(1):104–112. https://doi.org/10.1080/0886022X.2019.1583113

Since publication, significant concerns have been raised regarding the alleged duplication of images within Figure 1(C–E) and Figure 3(B,C) with those from other articles, specifically:

- Figure 1(B) from Sun Z, Zheng L, Liu X, Xing W, Liu X. Sinomenine inhibits the growth of melanoma by enhancement of autophagy via PI3K/AKT/mTOR inhibition. Drug Des Devel Ther. 2018;12:2413–2421. doi: https://doi.org/10.2147/DDDT.S155798.
- Figure 2(A) from Zhu H, Zhong S, Yan H, Wang K, Chen L, Zhou M, Li Y. Resveratrol reverts streptozotocin-induced diabetic nephropathy. Front Biosci. 2020;25(4):699–709. doi: https://doi.org/10.2741/4829. PMID: 31585912.
- Figure 2(E) from Zhang X, Gao F, Zhou L, Wang H, Shi G, Tan X. UCA1 regulates the growth and metastasis of pancreatic cancer by sponging miR-135a. Oncol Res. 2017;25(9):1529–1541. doi: https://doi.org/10.3727/096504017X14888987683152.
- Figure 2(G) from Gao F, Wang FG, Liu RR, Xue F, Zhang J, Xu GQ, Bi JH, Meng Z, Huo R. [Retracted] Epigenetic silencing of miR 130a ameliorates hemangioma by targeting tissue factor pathway inhibitor 2 through FAK/PI3K/Rac1/mdm2 signaling. Int J Oncol. 2020;57(6):1383. doi: https://doi.org/10.3892/ijo.2020.5136.
- Figure 4(A,B) from Fisher L. Retraction: overexpression of PCDH8 inhibits proliferation and invasion, and induces apoptosis in papillary thyroid cancer cells. RSC Adv. 2021;11(7):4163. doi: https://doi.org/10.1039/d1ra90011k.
- Figure 5(G) from Sun Y, Li L, Wu J, Gong B, Liu H. [Retracted] Germacrone cooperates with dexmedetomidine to alleviate high fat diet induced type 2 diabetes mellitus via upregulating AMPKα1 expression. Exp Ther Med. 2024;27(3):119. doi: https://doi.org/10.3892/etm.2024.12406.
- Figure 8(E) from Li Y, Zhu G, Ma Y, Qu H. LncRNA CCAT1 contributes to the growth and invasion of gastric cancer via targeting miR-219-1. J Cell Biochem. 2017. doi: https://doi.org/10.1002/jcb.26560.
- Figure 7(D) from Zhu L, Zhang Q, Li S, Jiang S, Cui J, Dang G. Interference of the long noncoding RNA CDKN2B-AS1 upregulates miR-181a-5p/TGFβI axis to restrain the metastasis and promote apoptosis and senescence of cervical cancer cells. Cancer Med. 2019;8(4):1721–1730. doi: https://doi.org/10.1002/cam4.2040. Retraction in: Cancer Med. 2024;13(1):e6713. doi: https://doi.org/10.1002/cam4.6713.

When asked for an explanation regarding the image similarities, the authors did not provide an explanation. However, the authors responded to request retraction due to issues with the data management process. No further information was provided regarding the data management issues.

As verifying the validity of published work is core to the integrity of the scholarly record, we are therefore, retracting the article. The corresponding author listed in this publication has been informed. The authors agree with the retraction.

We have been informed in our decision-making by our editorial policies and the COPE guidelines.

The retracted article will remain online to maintain the scholarly record, but it will be digitally watermarked on each page as ‘Retracted’.

© 2025 Informa UK Limited, trading as Taylor & Francis Group/Journal owner

